# Bayesian Analysis Using a Simple Likelihood Model Outperforms Parsimony for Estimation of Phylogeny from Discrete Morphological Data

**DOI:** 10.1371/journal.pone.0109210

**Published:** 2014-10-03

**Authors:** April M. Wright, David M. Hillis

**Affiliations:** Department of Integrative Biology, University of Texas at Austin, Austin, Texas, United States of America; British Columbia Centre for Excellence in HIV/AIDS, Canada

## Abstract

Despite the introduction of likelihood-based methods for estimating phylogenetic trees from phenotypic data, parsimony remains the most widely-used optimality criterion for building trees from discrete morphological data. However, it has been known for decades that there are regions of solution space in which parsimony is a poor estimator of tree topology. Numerous software implementations of likelihood-based models for the estimation of phylogeny from discrete morphological data exist, especially for the Mk model of discrete character evolution. Here we explore the efficacy of Bayesian estimation of phylogeny, using the Mk model, under conditions that are commonly encountered in paleontological studies. Using simulated data, we describe the relative performances of parsimony and the Mk model under a range of realistic conditions that include common scenarios of missing data and rate heterogeneity.

## Introduction

For many decades, parsimony methods have been the most widely used approaches for estimation of phylogeny from discrete phenotypic data, despite the availability of likelihood-based methods for phylogenetic analysis. Maximum likelihood and Bayesian methods are commonly used in data sets combining molecules and morphology [1]–[5], but are used less frequently in morphology-only data sets [6]. As such, the efficacy of these methods under a range of conditions is not well-explored. In particular, the conditions that are investigated in most paleontological studies (many characters missing across sampled taxa, and rate heterogeneity among different sampled characters) lead some investigators to raise questions about the applicability of model-based approaches under these conditions [6]–[9].

At the present, the most widely implemented (in both pure likelihood and Bayesian contexts) model for estimating phylogenetic trees from discrete phenotypic data is the Mk model proposed by Lewis [10]. This model is a generalization of the 1969 Jukes-Cantor model of nucleotide sequence evolution [11]. The Mk model assumes a Markov process for character change, allowing for multiple character-state changes along a single branch. The probability of change in this model is symmetrical; in other words, the probability of changing from one state to another is the same as change in the reverse direction. This assumption can be relaxed in Bayesian implementations through the use of a hyperprior allowing variable change probabilities among states [12]–[14]. As many morphologists collect only variable or parsimony-informative characters (i.e., characters that can be used to discriminate among different tree topologies under the parsimony criterion), the distribution of characters collected does not reflect the distribution of all observable characters. This sampling bias can lead to poor estimation of the rate of character evolution within a data set, as well as inflated estimates of character change along branches of the estimated tree. To counteract this bias, Lewis [10] introduced versions of the Mk model that correct for biases in character collection. These versions were subsequently shown to have the desirable quality of statistical consistency [15].

Sampled characters within data sets typically evolve under different rates, developmental processes, and modes of evolution [7], [16], [17]. Although heterogeneity in the underlying evolutionary processes can present challenges to the application of evolutionary models [18], a distribution of different evolutionary rates of characters can be helpful for resolving branches at different levels in the tree. Extremely labile characters, for example, are useful for resolving recently diverged lineages, whereas slowly evolving characters may be more useful for resolving deep divergences in the tree. Likelihood-based methods can benefit from this heterogeneity by accounting for different rates of character evolution and the amount of time available for change (based on the estimated branch lengths in the tree; [19]). In contrast, high levels of rate heterogeneity among characters can be more problematic for parsimony methods, especially if all character changes are weighted equally [20].

The ability to estimate branch lengths in numbers of changes per site or character is also useful for estimating divergence times. The Mk model, for example, is implemented in the software packages BEAST [21] and MrBayes [12], [13], [22] for use in divergence dating. Trees with explicit divergence dates are useful for a variety of comparative methods for answering evolutionary questions at a large scale. Methods for time-scaling parsimony trees and quantifying the uncertainty of these scaling methods exist [23], [24], [25], although at present, there is no thorough comparison of the performance of maximum likelihood, Bayesian, and parsimony-based approaches for morphological data.

Though there are many positive aspects of the Mk model (statistical consistency, ability to accept superimposed changes, explicit modeling of rate heterogeneity with a gamma distribution), paleontologists have been slow to adopt model-based approaches. Comparisons between the Mk model and parsimony analyses have provided interesting and illuminating results. For example, Xu et al. [26] found a controversial result when they added a new fossil taxon to an existing theropod data set and reanalyzed this expanded data set using parsimony. The reanalysis by Xu et al. supported a grouping of *Archeoptyeryx* with deinychosaurians—a change that has broad implications for the evolution of flight. In contrast, a further reanalysis of this data set with the Mk model by Lee and Worthy [6] yielded trees in which *Archeopteryx* was grouped in a more traditional placement with birds. An analysis of the characters supporting each topology demonstrated that the parsimony tree tended to be supported by characters with low consistency indices [6]. The Mk model has also been applied in co-estimation of phylogeny and divergence dates using fossils as terminal taxa in combined molecular–morphological data sets by several authors [22], [27], [28].

Here, we investigate the relative performance of parsimony and Bayesian analyses using the Mk model, under a variety of conditions applicable to paleontological investigations. We based simulations on empirically estimated trees so that we could sample realistic branch lengths and tree topologies. We then designed the simulations to investigate a range of factors associated with accuracy of phylogenetic estimation, including missing data, rate heterogeneity, and overall character change rate.

## Methods

### Simulations

To investigate the efficacy of the Mk model for phylogenetic estimation, we simulated data sets in the R package GEIGER [29]. We simulated characters under the discrete model of evolution—a modification of the Juke–Cantor model [11] for binary characters. Under this continuous-time Markov process, characters are simulated under a user-specified rate of change per character. For the single-rate data sets, one rate was drawn from a gamma distribution, and all characters were simulated according to this rate. For data sets with rate heterogeneity, each character had a rate of change drawn independently from the same gamma distribution. This approximates a condition under which each character has an independent evolutionary rate, which can be binned into discrete rates during phylogenetic analyses.

**Figure 1:**
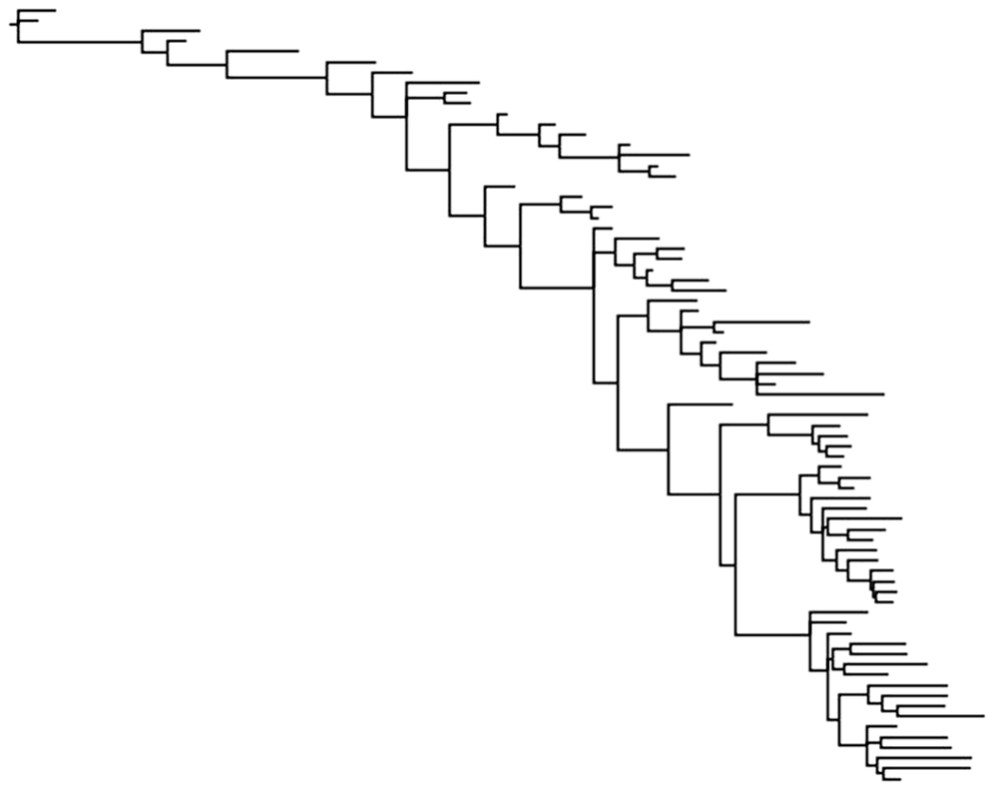
Tree used for simulations. This tree was obtained from a combined molecular–phenotypic data set analyzed by Pyron [27].

We simulated data sets of two sizes. The first data set size was 350 characters. This number of characters is representative for data sets of phenotypic data, as many published data sets are this size or smaller. We also simulated comparatively larger data sets of 1000 characters to investigate the effects of character sample sizes. The empirical tree along which data were simulated was based on the tree presented by Pyron [27] and was chosen for its complexity. This tree (Figure 1) contains many short branches, which is representative of many analyses that include fossil specimens.

### Ascertainment bias in morphological characters

Phenotypic data are often filtered by an observer-defined scheme. Characters that do not vary or vary in a parsimony uninformative way (such as autapomorphies) are usually excluded from analysis. In contrast to molecular sequence data, this means that there are rarely invariant sites in paleontological data sets. This bias can result in inflation of the estimated rate of evolutionary change in the data set, increasing the estimated branch lengths on the tree [10]. Under likelihood-based methods, branch lengths are estimated alongside tree structure, and unrealistically-inflated branch lengths can lead to topological error. MrBayes incorporates three versions of the Mk model. The uncorrected model (Mk) does not account for any form of sampling bias. Two corrected models account for the bias of collecting only variable characters (Mkv) and the bias of collecting only parsimony-informative characters (Mk-pars). To examine the effects of character acquisition bias, we filtered data sets according to different data acquisition schemes. The unfiltered data sets contained invariant characters, variable characters that were not parsimony-informative (e.g., autapomorphies), and variable characters that were parsimony-informative. Intermediate data sets excluded invariant sites, but retained variable sites that were not parsimony-informative. The least inclusive data sets contained only parsimony-informative characters.

Each character filtration scheme was parameterized appropriately in MrBayes. We did not explore the effects of model misspecification or incorrectly accounting for acquisition bias in this study. Data files can be found in the online supporting material, along with scripts for assembling MrBayes and PAUP blocks.

### Missing Data

**Figure 2:**
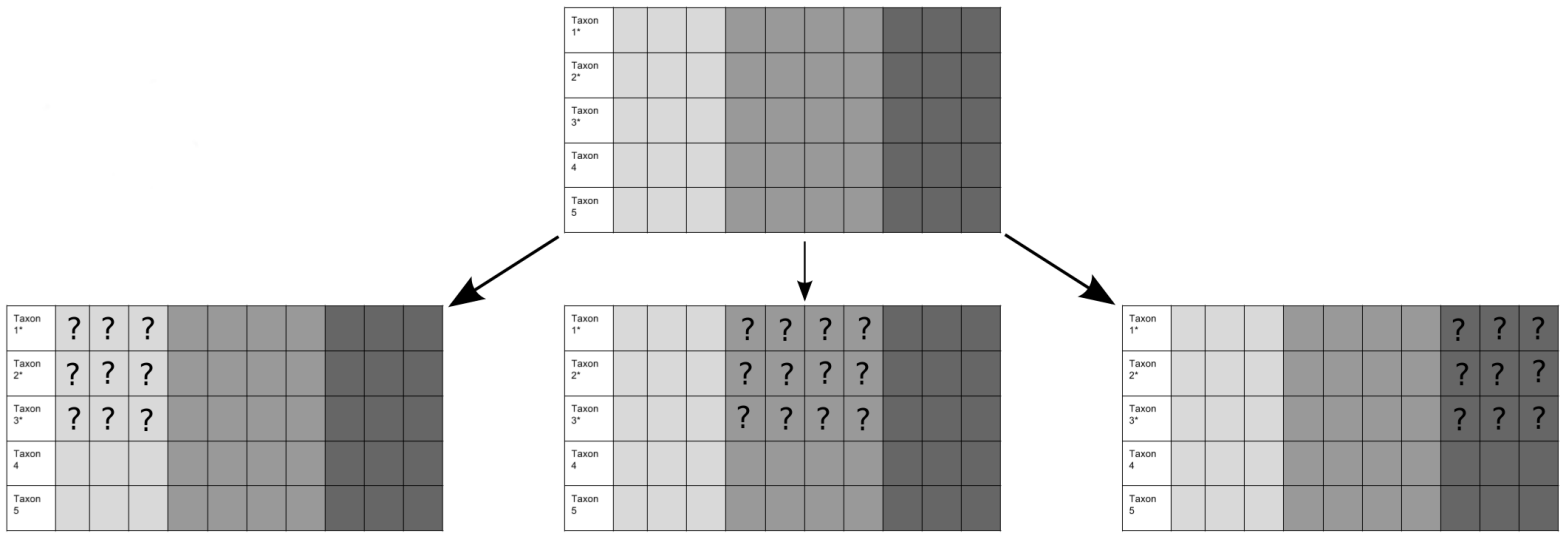
A schematic representing different missing data distributions. Columns represent characters. In the taxon-names column, an asterisk represents fossil taxa. Characters with the slowest rate of change are represented in light grey; intermediate-rate characters are represented in medium grey; characters with highest rate of change are represented in dark grey. In the top matrix, all characters are present for all taxa. The bottom matrices illustrate the missing data conditions that we simulated in this paper.

To assess the effects of missing data on phylogenetic estimation, we used several schemes for character deletion. We sorted the characters by rate of change, and divided them into three categories: fast-, intermediate-, and slow-evolving sites. Within each class of sites, we created data sets in which we removed between 10% and 100% of sites to investigate the effects of underrepresentation of certain classes of characters. Missing data were concentrated in fossil taxa, as seen in Figure 2.

### Estimating Phylogenetic trees

We estimated Bayesian phylogenetic trees in MrBayes 3.2.2 [12], [13] on the Lonestar server of the TACC computing facility at the University of Texas–Austin. We used the majority-rule consensus tree returned by MrBayes in all calculations and comparisons.

We used PAUP* for parsimony analyses. In PAUP* [30], we estimated phylogenetic trees using the TBR swapping algorithm with random branch addition and one thousand replicates. Estimation was performed on a ROCKS v4.1 computing cluster.

### Analysis of Estimated Trees

There are many ways to categorize how well a tree has been estimated. Given that these data were simulated under a tree, we can compare the estimated phylogenetic trees to the true phylogenetic tree. We used a script written in Python, making use of the Dendropy library [31], to calculate the symmetric distance (the unweighted Robinson–Foulds distance [32]) between the estimated trees and the phylogenetic tree under which the data were generated. For unrooted trees of *N* taxa, there are *N*–3 bipartitions of the taxa (excluding bipartitions involving single taxa, which are the same for all trees). The Robinson–Foulds distance considers both the presence of incorrect bipartitions as well as the absence of correct bipartitions, so the maximum symmetric distance between two trees is 2(*N*–3). Therefore, for a 75-taxon tree, the maximum Robinson–Foulds distance is 144 symmetric distance units. For ease of interpreting graphs, we rescaled these values so that the total error is 100% (which would indicate all bipartitions in the tree are estimated incorrectly).

In a Bayesian analysis, the posterior sample of trees is not comprised of equally optimal solutions. Instead, each tree in the sample typically has a different likelihood score. A majority-rule consensus tree can be used to summarize the variation across the posterior sample, and this consensus tree is often taken as a summary estimate of the phylogeny. Therefore, we used the symmetric distance from the majority-rule consensus tree of the posterior sample to the model tree to evaluate the performance of the Bayesian analyses. In contrast, under the parsimony criterion, equally parsimonious trees are each considered optimal alternative solutions. Therefore, in parsimony analyses, we calculated the symmetric distance from each equally parsimonious solution to the model tree, and then averaged these scores within each data set to obtain an average symmetric distance score. We also used a majority-rule consensus tree to evaluate the parsimony analyses, and found the results were almost identical with the two measures (Fig. S2). All code to replicate results can be found in the online Supplemental Information.

## Results

### Character Filtration

Sampling bias does not affect Bayesian estimation when appropriate corrections are implemented. Correcting for ascertainment bias in MrBayes [12]–[13] is described by Lewis [10] based on the unobserved character counting method of Felsenstein [33]. In this approach, a likelihood for the data set is calculated conditional on only variable or parsimony informative characters present in the data. This conditional likelihood is then combined with the likelihood of a hypothetical constant character to arrive at a correction for acquisition bias. As shown in Figure S1, all parameterizations of the Mk model in MrBayes returned the same distributions of error. This demonstrates that corrections for sampling schemes are effective.

### Single-Rate Simulations

**Figure 3:**
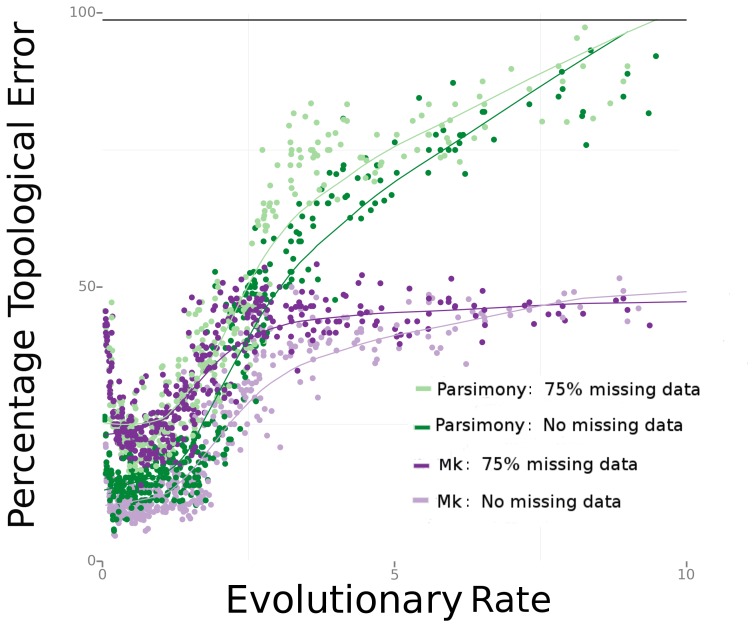
Results from simulations with a single rate of character evolution. Bayesian-Mk outperforms parsimony most strongly when the rate of character evolution (and hence homoplasy) is high.

As seen in Figure 3, at the lowest evolutionary rates, the amount of error in phylogenetic trees estimated compared to the true tree is fairly high, with nearly one in five branches being incorrectly estimated for both Bayesian and parsimony estimation. We would expect this to be true, as in this region of the graph, there are few character changes in the matrix. As evolutionary rate is increased, topological error reaches a minimum in error for both types of estimation. This minimum occurs at about one expected change per character. As more changes per character occur, there is an increase in topological error. This increase in error is seen more sharply in parsimony than Bayesian estimation, as Bayesian methods account for superimposed and parallel changes. Among different corrections of the Mk model for acquisition bias, performance is very similar (Figure S1).

As the amount of missing data increases in these data sets, the amount of error also increases. With 75% of data missing, as seen on Figure 3, parsimony and the Bayesian implementation of the Mk model perform very similarly at low rates of character change. However, at high rates of character change, the Bayesian Mk method outperforms parsimony strongly. In these regions of sample space, the characters show a poorer fit to the tree, with many characters exhibiting parallelisms and reversals.

### Rate Heterogeneity

**Figure 4:**
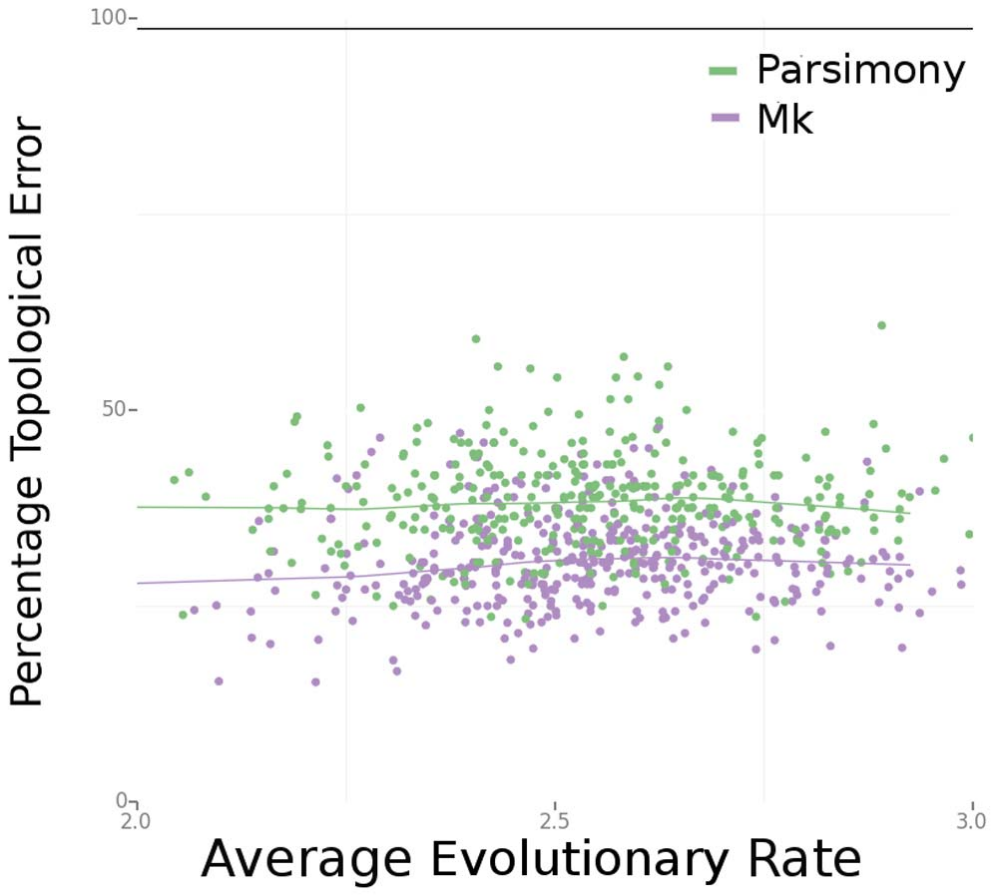
In data sets with character rate heterogeneity and with no missing data, Bayesian-Mk results in lower error compared to parsimony analyses. Note that, unlike Figure 3, the X-axis is the average rate of change across all characters in the data set, as opposed to one single rate applied uniformly to all characters.

**Figure 5:**
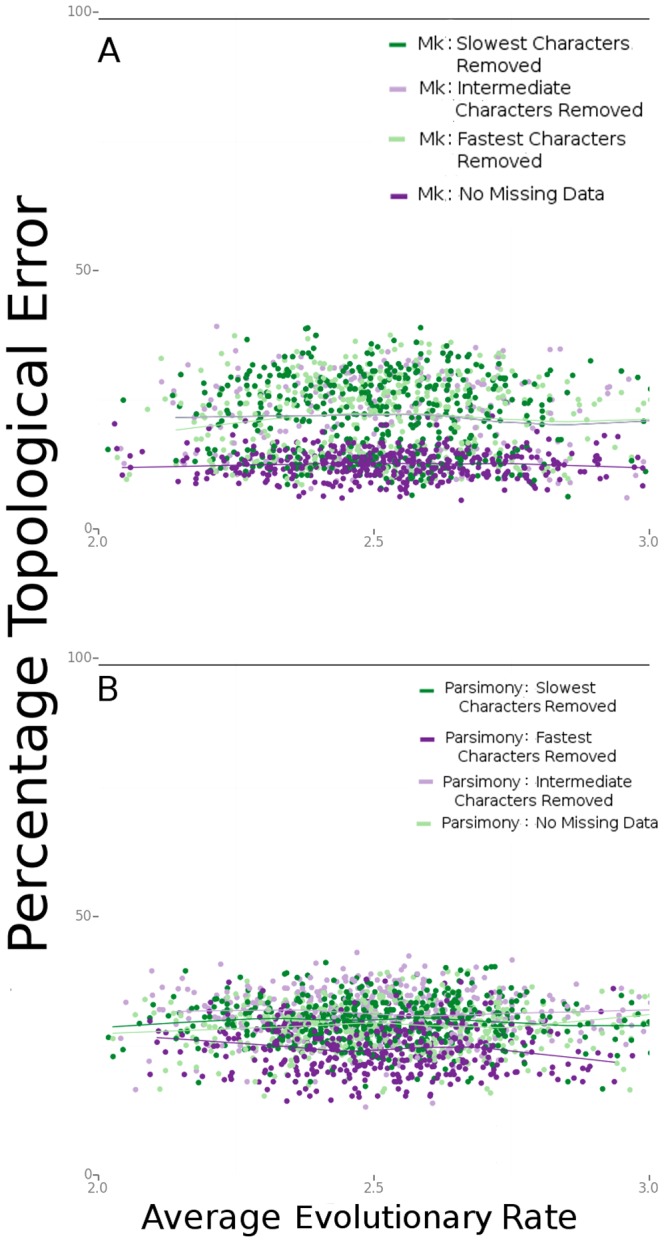
The effects of missing data vary with the rate of character evolution. This figure compares the effect of deleting one-third of the characters from three different rate classes. (A) Comparisons of Bayesian-Mk analyses. (B) Comparisons of parsimony analyses.

In data sets with rate heterogeneity among the characters, the Mk model continues to outperform parsimony, as shown in Figure 4. We also examined the effects of structured missing data in these data sets. Figure 5 compares the effects of removing various classes of characters (of different evolutionary rates) in the Bayesian Mk and parsimony analyses.

**Figure 6:**
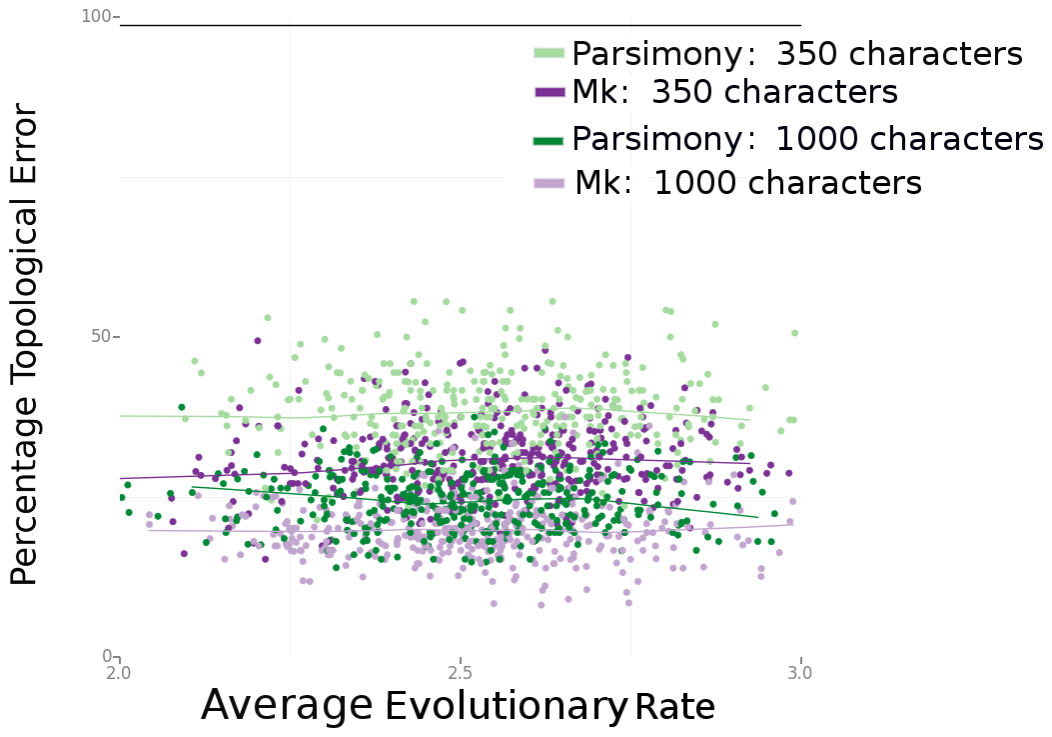
Comparison of 350- and 1000-character data sets.

Both Bayesian Mk analyses and parsimony show degraded performance when characters of different rate classes are removed from the analysis, although the negative effects of missing data are much greater for parsimony than for the Bayesian analyses (especially for deletion of the slowest-evolving characters). Part of this effect is related to reduction in the overall number of characters available for analysis. Increasing the total number of characters in the analysis improves the performance for both Bayesian and parsimony analyses, although the Bayesian analyses continue to exhibit higher accuracy compared to parsimony in the 1000-character analyses (Figure 6).

## Discussion

Our results suggest that Bayesian methods of analysis are likely to exhibit lower error rates compared to parsimony analyses in phylogenetic analyses of morphological and paleontological data sets. Moreover, researchers should carefully consider character-sampling design, as error rates can increase if characters are evolving too rapidly (Figure 3). As seen in Figure 3, before missing data or rate heterogeneity are introduced, phylogenetic estimation is most accurate for characters with relatively slow rates of change, as long as they are evolving fast enough to produce some phylogenetic signal. In these regions of the sample space, parsimony and Bayesian methods perform very similarly.

However, it is unlikely that empirical data sets will have only one rate of evolution across the whole data set. Rather, they are likely to be made up of characters that have been subjected to different selective pressures, different developmental constraints, and different evolutionary processes [16], [17]. Rate heterogeneity in morphological data sets is well documented [7]. Therefore, the relationship between topological error and the location of missing data within a data set is of interest to researchers who build trees, as systematically under-representing certain classes of characters may produce different effects. Slowly-evolving characters include some characters that have too little change to be parsimony-informative; the fastest-evolving characters in these data sets include some characters with reversals and parallelism. In likelihood-based analyses, both parsimony-noninformative and parsimony-misinformative characters are still useful, as they provide information about the average rate of evolution in a data set. Rapidly-evolving characters can mislead parsimony analyses, which are unable to account for superimposed changes on a given branch. It would be expected that removing slowly-evolving characters (even those that are not parsimony-informative) would inflate the estimated average evolutionary rate, potentially leading to branch-length overestimation, and removing characters that change many times on the tree would result in underestimation of the average evolutionary rate. Figure 5 supports this conclusion, demonstrating that removing either of these classes of characters does result in higher topological error. Removing any class of characters (but especially the slowest-evolving characters) also results in lower performance of the parsimony analyses (Figure 5), presumably due to loss of information in an already small data set. Concerns about missing data have been cited as a reason to choose parsimony over likelihood-based methods [9]. Our results suggest that incomplete matrices do not necessitate the use of parsimony.

Increasing the size of the data set improves estimation for both parsimony and Bayesian methods. However, even in large data sets with no missing data, the Bayesian analyses using a simple likelihood model of character change typically outperform parsimony analyses (Figure 4). Paleontologists may be strongly constrained in how many characters or taxa they can add to a data set, due to a lack of specimens, a lack of observed homologous characters across a clade of interest, or poor specimen quality. Our results suggest that the use of Bayesian methods is even more important when relatively few characters are analyzed, and that even a simple probabilistic model can considerably improve the accuracy of tree estimation.

The benefits of adding fossil taxa to a data set are numerous. Earlier research has argued that fossil taxa can alleviate the issue of long-branch attraction (LBA), particularly when additional extant taxa cannot be added to break up long branches [34], [35]. Previous simulations have also suggested that, in combined analysis, even highly incomplete fossils can help alleviate the affects of LBA [36]. Empirical studies have confirmed these results, indicating that fossils with up to 75% missing data can help improve resolution in parsimony analysis [37] and result in vastly different topologies compared to molecular-only analyses [38]. Our results indicate that a model-based analysis is an even more effective way to gain performance improvements from such additions of fossil taxa.

In addition to exhibiting lower error rates, model-based methods offer another important advantage over parsimony: the ability to estimate time based on branch lengths of the phylogenetic tree. The Mk model, for example, is implemented in the software packages BEAST [21] and MrBayes [22] for use in divergence dating (although in BEAST, characters that are not variable or parsimony-informative must be explicitly listed by the author; see [38] for a discussion of counting unobserved site patterns). In turn, trees with explicit divergence dates are useful with a variety of comparative methods [39]. Methods for time-scaling parsimony trees exist [24], [40], [41], [42], although at the present, there is no thorough investigation of the performance of model-based versus parsimony-based approaches for estimating time with morphological data.

Our results demonstrate that Bayesian methods are more accurate than parsimony for estimating trees from discrete morphological data under a wide set of realistic conditions. Even when there are large amounts of missing data (as is common in paleontological studies), a simple likelihood model consistently produces less error in tree estimation compared to parsimony. Although there is considerable room for models of morphological character evolution to be improved, even simple model-based methods can result in considerable improvement of phylogenetic analyses of morphological data sets.

## Supporting Information

Figure S1 **The effect of filtering characters before estimating phylogenies in a Bayesian context.** MrBayes has three parameterizations of the Mk model, which account for sampling bias. As seen above, these methods estimate trees with the same degree of accuracy under the conditions we examined.
(TIFF)

Figure S2 **Parsimony analyses return sets of equally optimal trees.** A symmetric difference score to the true (model) tree can be calculated either by creating a consensus tree and using this tree to calculate the symmetric difference, or by calculating the symmetric difference for every tree in the solution set and averaging this score. In our study, these two methods produce very similar results.
(TIFF)
