# Supplementary figures and images for: Bayesian Analysis Using a Simple Likelihood Model Outperforms Parsimony for Estimation of Phylogeny from Discrete Morphological Data
Source: PLoS One. 2014 Oct 3;9(10):e109210. doi: 10.1371/journal.pone.0109210 (PMC4184849; doi:10.1371/journal.pone.0109210)

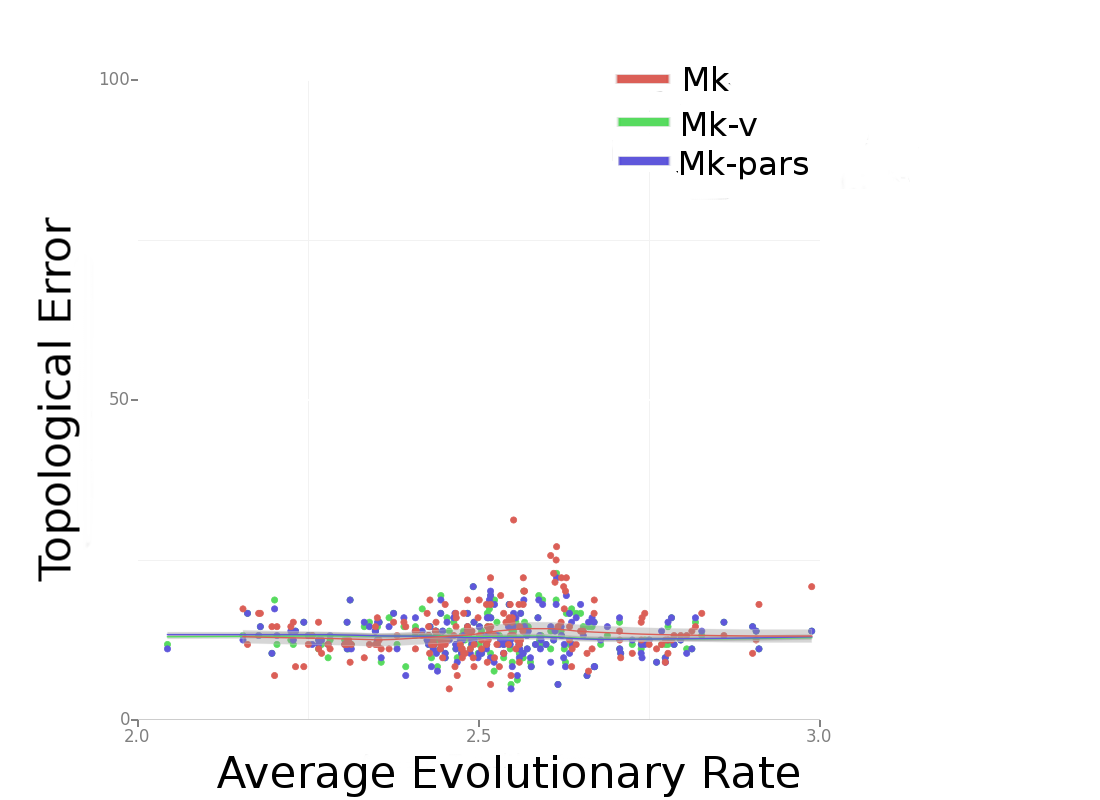

Supplement: Figure S1 — The effect of filtering characters before estimating phylogenies in a Bayesian context. MrBayes has three parameterizations of the Mk model, which account for sampling bias. As seen above, these methods estimate trees with the same degree of accuracy under the conditions we examined. (TIFF) [file pone.0109210.s001.tiff]

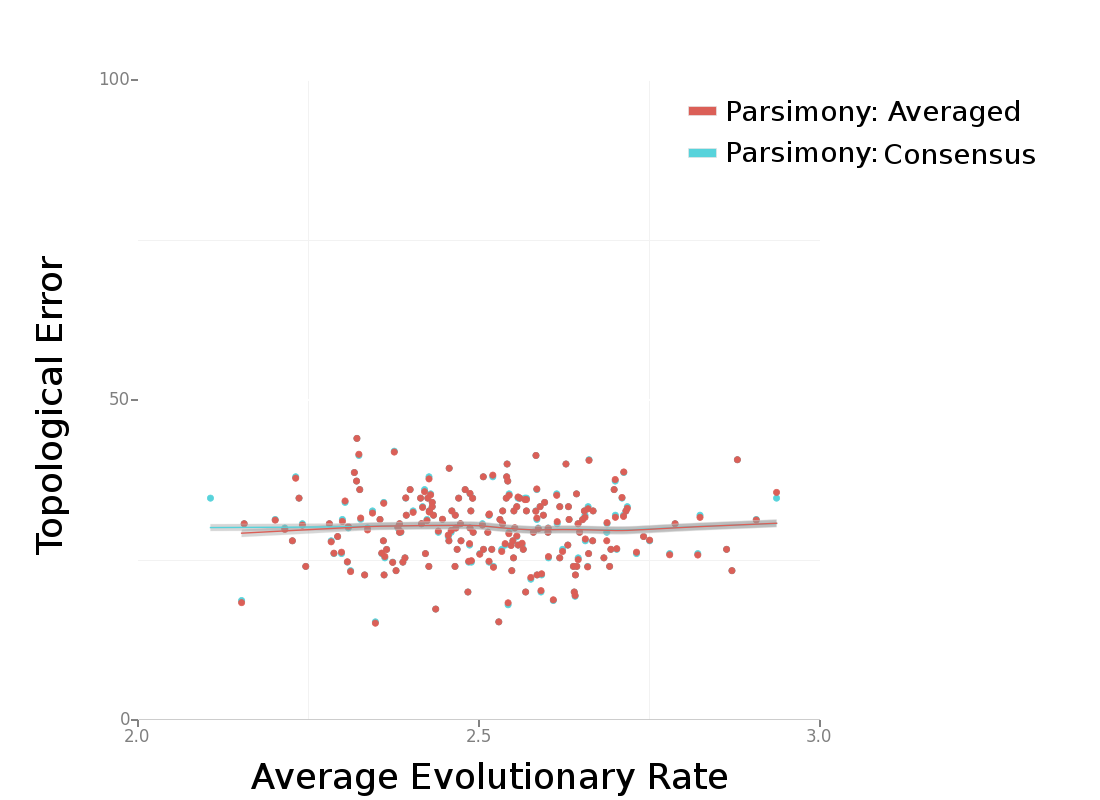

Supplement: Figure S2 — Parsimony analyses return sets of equally optimal trees. A symmetric difference score to the true (model) tree can be calculated either by creating a consensus tree and using this tree to calculate the symmetric difference, or by calculating the symmetric difference for every tree in the solution set and averaging this score. In our study, these two methods produce very similar results. (TIFF) [file pone.0109210.s002.tiff]
